# Characterization of a Novel Double-Stranded RNA Virus from *Phytophthora pluvialis* in New Zealand

**DOI:** 10.3390/v14020247

**Published:** 2022-01-26

**Authors:** Zhi Xu, Mahmoud E. Khalifa, Rebekah A. Frampton, Grant R. Smith, Rebecca L. McDougal, Robin M. MacDiarmid, Falk Kalamorz

**Affiliations:** 1 The New Zealand Institute for Plant and Food Research Limited, Canterbury Agriculture & Science Centre, 74 Gerald Street, Lincoln 7608, New Zealand; Zhi.Xu@plantandfood.co.nz (Z.X.); Rebekah.Frampton@plantandfood.co.nz (R.A.F.); Grant.Smith@plantandfood.co.nz (G.R.S.); 2 School of Biological Sciences, University of Auckland, Auckland 1142, New Zealand; Robin.MacDiarmid@plantandfood.co.nz; 3 The New Zealand Institute for Plant and Food Research Limited, 120 Mt Albert Road, Sandringham, Auckland 1025, New Zealand; mkha201@aucklanduni.ac.nz; 4 Scion, New Zealand Forest Research Institute, Ltd., 49 Sala Street, Te Papa Tipu Innovation Park, Rotorua 3010, New Zealand; Rebecca.McDougal@scionresearch.com

**Keywords:** virus, *Phytophthora pluvialis*, dsRNA, qPCR, New Zealand

## Abstract

A new dsRNA virus from the oomycete *Phytophthora pluvialis* has been characterized and designated as Phytophthora pluvialis RNA virus 1 (PplRV1). The genome of the PplRV1 reference genome is 6742 bp that encodes two predicted open reading frames (ORFs). ORF1 and ORF2 overlap by a 47 nt “slippery” frameshift sequence. ORF1 encodes a putative protein of unknown function. ORF2 shows high similarity to the RNA-dependent RNA polymerase (RdRp) of other dsRNA viruses. Phylogenetic analysis of the putative PplRV1 RdRp and its most closely related viruses showed PplRV1 is distinct from other known viruses (below 33% amino acid similarity), which indicates this virus may belong to a new virus family. Analyses of the geographical distribution of PplRV1 in relation to two genetically distinct classes of its host revealed two corresponding genotypes of the PplRV1 (termed a and b), which share 92.3% nt identity. The reference genome for the second genotype is 6760 bp long and a prediction of its genetic organization shows three ORFs, with ORF2 being split into two ORFs, ORF2a and ORF2b, that is conserved in seven of eleven genotype b isolates. Additionally, a quick and simple diagnostic method using qPCR has been developed, which is suitable for large scale screens to identify PplRV1 in *Phytophthora*.

## 1. Introduction

*Phytophthora* is a genus in the family *Oomycota*, which was previously classified within the fungi domain and separated owing to their highly divergent genome sequence [1]. Most *Phytophthora* species are plant pathogens and cause enormous economic losses in crops worldwide. The most notorious plant pathogen, *Phytophthora infestans*, causes potato late blight which in the 1840s triggered a series of events that led to several million people dying from starvation [2]. Another example is *Phytophthora agathidicida*, the causal agent of the devastating disease kauri dieback which threatens kauri tree (*Agathis australis*) populations of high ecological and cultural relevance in New Zealand [3,4,5].

*Phytophthora pluvialis* is a pathogen causing red needle cast disease. In 2008, a severe needle disease of *Pinus radiata* caused by a new and undescribed *Phytophthora* was reported in New Zealand [6]. In 2013, *P. pluvialis* was isolated from stream, soil and canopy drip in tanoak-Douglas fir forest in Oregon (USA) and described as a new pathogen species within the *Phytophthora* clade III [7]. A year later, *P. pluvialis* was first recognized in the North Island of New Zealand when an outbreak of red needle cast disease on pine trees (*Pinus radiata*) occurred [6,8].

A study using single nucleotide polymorphism (SNP) and a minimum spanning network identified two clusters of *P. pluvialis* isolates from New Zealand, which suggested *P. pluvialis* was potentially introduced from the USA into New Zealand on two separate occasions [9]. A subsequent comparison with isolates from the USA using a larger range of SNP markers showed that one of these New Zealand clusters is closely related to isolates from the USA, while the other may represent an evolutionary adaptation to the new environment [10].

Increasing numbers of viruses have been reported in *Phytophthora* since the first virus was identified in 2005. Originally, an endornavirus was isolated from a *Phytophthora* isolate from Douglas fir [11] and named Phytophthora endornavirus 1 (PEV1). The genome of PEV1 is 13,883 nt, and contains an open reading frame (ORF) encoding a potential RNA-dependent RNA polymerase (RdRp) [11]. PEV1 has not only been confirmed in a *Phytophthora* isolate from Douglas fir but also in *Phytophthora ramorum* [12]. In 2020, a dsRNA virus was discovered infecting *Phytophthora cactorum* and designated Phytophthora cactorum RNA virus 1 (PcRV1) [13]. Phylogenetic analysis reveals this virus is closely related to the unclassified Pythium splendens RNA virus 1, which is provisionally classified into the virus family *Totiviridae* [14]. To date there are seven virus species recognized within *Phytophthora* [11,12,13,15,16,17,18].

Several RNA viruses have been found in single *Phytophthora* species. For example, within *Phytophthora infestans* alone four viruses have been described. An RNA virus was reported in *P. infestans* and designated Phytophthora infestans RNA virus 1 (PiRV-1) [15]. PiRV-1 has two RNA segments, RNA1 and RNA2, with a length of 3160 nt and 2730 nt, respectively. RNA1 encodes a hypothetical protein and an RdRp. RNA2 is predicted to encode a polyprotein and an ORF of unknown function. Phylogenetic analysis reveals PiRV-1 does not belong to any known virus family [15]. Another RNA virus, Phytophthora infestans RNA virus 2 (PiRV-2), was discovered in *P. infestans* in 2018 [18]. The genome of PiRV-2 is 11,170 nt, which contains a single predicted ORF spanning the nucleotides 7 to 11,139. It encodes a putative polyprotein that functions as an RdRp [18]. The positive sense, single strand RNA (ssRNA) virus Phytophthora infestans RNA 3 (PiRV-3) was first been reported in 2013 and has a 8112 nt long genome that has two predicted ORFs with a 31 bp overlap. The ORF1 of PiRV-3 potentially encodes a protein with an unknown function and ORF2 putatively encodes an RdRp [17]. In 2012, another virus named Phytophthora infestans RNA virus 4 (PiRV-4) was reported by the same group [16]. The genome of PiRV-4 is small, only 2984 nt. A single predicted ORF in the PiRV-4 genome potentially encodes an RdRp. A phylogenetic analysis of the PiRV-4 RdRp shows it is related to the positive sense ssRNA narnaviruses [16].

Beside RNA viruses, DNA viruses have also been reported in *Phytophthora* species. In 2011, Liu et al. found gemini-like sequences integrated into the mitochondrial genomes of *P. infestans* and *Phytophthora sojae* [19]. The sequence integrated into the *P. infestans* mitogenome shares approximately 70% similarity with the one found in *P. sojae*. It has been assumed that the replication-initiation sequences of some circular DNA viruses, such as geminiviruses, are frequently transferred into a wide variety of eukaryotic hosts [19]. Furthermore, several of the integrated genes are maintained and expressed in the host genomes [19].

Viruses or viral-like sequences can contribute to host-adaptive phenotypes, including thermal tolerance and virulence. For example, *Dichanthelium lanuginosum*, a tropical panic grass, colonized by the fungus *Curvularia protuberata* which itself is infected with the *C. protuberata* thermal tolerance virus, is more heat-tolerant than plants harboring virus-free *C. protuberata* [20]. A group of mycoviruses were found to be able to decrease the infectivity of their pathogenic fungal host, such as Cryphonectria hypovirus 1 (CHV1) infesting *Cryphonectria parasitica*, which causes chestnut blight [21,22]. Because CHV1 is also associated with an increase in the survival time of host plants, this mycovirus was used as a biocontrol agent of chestnut blight in Europe [21]. Additionally, study revealed PiRV-2 affects the sporulation in *P. infestans* [23]. Compared to PiRV-2-free isogenic isolates, *P. infestans* isolates that contained PiRV-2 produced a larger number sporangia. Further surveys found PiRV-2 was present in most strains of a predominant clonal lineage of *P. infestans* in North America, which suggested PiRV-2 could be the cause of the resurgence of potato late blight in the 1980s–1990s [23]. Mycoviruses that reduce the pathogenicity of their fungal host are known as hypoviruses. They have the ability to decrease the chance of infection, colonization and/or death by the pathogenic fungus. Conversely, viruses that increase the infectivity of fungi are called hyperviruses and these are typically associated with reduced survival time of infected plants [24].

Understanding of the potential impacts of *Phytophthora* viruses on *Phytophthora*, either hypervirulence or hypovirulence, may lead to alternative options to control pathogenic *Phytophthora*. This study discovered and characterized an RNA virus associated with New Zealand *P. pluvialis*, which provides fundamental knowledge and may identify leads for developing a biocontrol method to manage pathogenic *P. pluvialis*.

## 2. Methods

### 2.1. Initial Screen for Phytophthora dsRNA Elements

An initial screen for double-stranded RNA elements was performed with isolates NZFS 4014 to 4019 collected in Gisborne, New Zealand, using the CF-11 cellulose chromatography protocol [25], with subsequent analysis on 1% SDS-Agarose gels. The extracted dsRNA was purified as described by Valverde et al. (1990). Illumina sequencing and bioinformatics analysis were then performed as described in Khalifa et al. (2016).

### 2.2. P. pluvialis Isolates for Virus Genome Characterization

*P. pluvialis* isolates collected in the New Zealand regions of Auckland (n = 1), Bay of Plenty/Wanganui (n = 1), Coromandel (n = 2), Gisborne (n = 7), Hawke’s Bay (n = 1), Nelson (n = 3), Northland (n = 5), Taranaki (n = 1), Taupo (n = 1) and Wairarapa (n = 1) were obtained from the New Zealand Forest Research Institute (Scion) (Rotorua, New Zealand). Among these *P. pluvialis*, two are isolates whose cluster is unknown, 12 isolates belong to New Zealand cluster 1 (NZ1), and nine isolates belong to New Zealand cluster 2 (NZ2) [9]. Moreover, one isolate was isolated from Douglas-fir (*Pseudotsuga menziesii*), and 22 isolates were sourced from radiata pine (*Pinus radiata*) (Table S1). Isolates were maintained on V8 agar [26] supplemented with 250 mg/L ampicillin (AppliChem GmbH, Darmstadt, Germany), 10 mg/L natamycin (Sigma-Aldrich^®^, St. Louis, MO, USA) and 250 mg/L rifampicin (Sigma-Aldrich^®^, St. Louis, MO, USA) at room temperature in the dark.

### 2.3. dsRNA Extraction and Sequencing

After 12–14 days, 100–400 mg *Phytophthora* mycelia were collected for dsRNA extraction. Mycelia were ground in liquid nitrogen using a sterilized pestle and mortar. The dsRNA was extracted using Double-RNA Viral dsRNA Extraction Mini Kit (ABC Scientific^®^, Glendale, CA, USA) according to the manufacturer’s instructions for plant tissue.

Virus dsRNA was reverse-transcribed into complementary DNA (cDNA) with primer SEQ31 designed based on the viral sequences initially isolated using SuperScript™ IV First-Strand cDNA Synthesis Reaction Kit (Invitrogen, Carlsbad, CA, USA). Subsequently, it was amplified via Polymerase chain reaction (PCR) with primers SEQ31 and SEQ6663 (Table S2). The full-length sequence of this cDNA was obtained by primer walking and 5′ rapid amplification of cDNA ends (5′ RACE). Virus genomes from isolates NZFS 3052 and NZFS 4018 were chosen as reference for PplRV1 from *P. pluvialis* clusters NZ1 and NZ2, respectively [10]. To obtain the terminal sequences, 5′ RACE was performed on virus dsRNA. Virus dsRNA was reverse-transcribed into cDNA by reverse transcriptase (RT) and the RACE1 primer (Table S2). A poly (A) tail was then added to the cDNA ends using terminal deoxynucleotide transferase (New England Biolabs, Ipswich, MA, USA). The polyadenylated cDNA fragment was amplified by the anchor primer and primer RACE2. Terminal sequences were finally amplified by the adaptor primer and primer RACE3. The size and quality of the resulting products were checked by 1% agarose gel electrophoresis and NanoDrop™ 1000 Spectrophotometer (Thermo Fisher Scientific, Madison, WI, USA).

The purified PCR products were sequenced via a commercial Sanger sequencing service (Massey University Genome Sequencing Service, Palmerston North, New Zealand). Subsequently a sequencing strategy using eight distinct overlapping PCR was established (see Table S2 for details). Virus dsRNA from 23 *P. pluvialis* isolates were fully/near completely sequenced.

### 2.4. Bioinformatic Analysis

Low-quality ends of the Sanger sequences amplified were trimmed and *de novo* assembled using Geneious 10.0.9 (Biomatters, Auckland, New Zealand). The sequence assembly were aligned to the preliminary contigs 1 and 2 assembled from the initial screen. The ORFs in the virus genome were predicted by Geneious 10.0.9.

Virus genomes from NZFS 3052 and NZFS 4018 were used as the references for virus sequence assembly from additional *P. pluvialis* isolates. Virus genomes from *P. pluvialis* isolates belonging to NZ1 and NZ2 [10] were aligned using MAFFT [27,28] via Geneious 10.0.9 to identify any cluster-specific single-nucleotide polymorphisms (SNPs). The nucleotide alignment were used to build a phylogenetic trees using PhyML 3.0 [29,30] via Geneious 10.0.9.

A conserved region within the virus genome was found through NCBI Conserved Domain Database [31] searches. BLASTx search of the predicted conserved region was performed. Amino acid sequences of the putative conserved region and its related viruses, including Wuhan insect virus 28 (accession: YP_009342430), Circulifer tenellus virus 1 (accession: YP_003800003), Spissistilus festinus virus 1 (accession: YP_003800001), Culex vishnui subgroup totivirus (accession: BBQ05098), Stinn virus (accession: QRW41701), Koroku virus (accession: QRW41695), Hubei toti-like virus 10 (accession: YP_009336493), Phytophthora infestans RNA virus 3 (accession: YP_009551328) and Bremia lactucae associated fusagravirus1 (accession: QIP68009) were then aligned using MAFFT [27,28]. Additionally, an alignment of the amino acid sequence of the putative conserved region within the virus genome was generated using Clustal Omega [32]. FSFinder 2 [33] was used to identify frameshifts within the virus genomes. The structure of the predicted slippery sequence was analyzed and visualized in Geneious 10.0.9.

### 2.5. Virus Diagnostics

A panel of *Phytophthora*/*Nothophytophthora* isolates was used for the virus screen, including 25 *P. pluvialis* isolates, nine other *Phytophthora* species/isolates and one *Nothophytophthora* species. The 25 *P. pluvialis* isolates were collected in Bay of Plenty (n = 2), Gisborne (n = 1), Hawke’s Bay (n = 3), Nelson (n = 5), Rangitikei (n = 3), Taranaki (n = 1), Taupo (n = 3), Waikato (n = 1), Wairarapa (n = 2), Wanganui (n = 1), Wellington (n = 3) in New Zealand. Two *P. cactorum* isolates were collected in the Bay of Plenty in New Zealand. Two *Phytophthora cryptogea* isolates were collected in the New Zealand regions of South Canterbury and Auckland. Two *Phytophthora multivora* isolates were collected in Auckland and the Bay of Plenty, and three *Phytophthora* hybrid isolates were collected in Auckland, the Bay of Plenty and Northland regions in New Zealand.

*Phytophthora* total RNA was extracted using RNeasy Mini Kit (Qiagen, Hilden, Germany) following the manufacturer’s instructions. The RT- quantitative PCR (RT-qPCR) primers and probes were designed by Primer3 [34,35] via Geneious 10.0.9. A duplex RT-qPCR with primers PplRV1_qPCR_F1 and PplRV1_qPCR_R1 and probe PplRV1_probe1 for PplRV1 detection combined with primers PhyG-F2 and PhyG-Rb and probe TrnM_PhyG_probe2 as host control was used for detecting PplRV1 (see Table S2 for details). The RT-qPCR was performed using iTaq Universal Probes One-step kit (Bio-Rad, Hercules, CA, USA). Annealing temperature for qPCR amplification was optimized by gradient qPCR, followed by primer and probe optimization. The final concentration of primers PplRV1_qPCR_F1 and PplRV1_qPCR_R1 and probe PplRV1_probe1 were 400 nM/400 nM and 250 nM, respectively. The final concentration of primers PhyG-F2 and PhyG-Rb and probe TrnM_PhyG_probe2 were 500 nM/500 nM and 250 nM, respectively. An aliquot (2 µL) of *Phytophthora* total RNA was used in each 20-µL reaction. The optimized reactions are summarized in Table S3. The RT-qPCR cycling followed the manufacturer’s instructions with the modification of annealing temperature at 55 °C. Total RNA was firstly reverse transcribed into cDNA at 50 °C for 10 min, followed by 1 min of polymerase activation and DNA denaturation at 95 °C. Amplification consisted of 40 cycles of 10 s of denaturation at 95 °C and 30 s of annealing then extension at 55 °C. Fluorescence was read following the annealing and extension step of each cycle. Data analysis was conducted using Bio-Rad CFX Maestro™ Software (Bio-Rad, Hercules, CA, USA). The specificity and sensitivity of the assay was established using a panel of 35 *Phytophthora*/*Nothophytophthora* strains (Table S4).

## 3. Results

### 3.1. Virus Sequence and Genome Characterisation

Initial screen for *Phytophthora* dsRNA elements identified three viral contigs. The three contigs were assembled from dsRNA-derived cDNA sequences and translated in silico: contig 1 (3395 nt) and contig 2 (2865 nt) shared approximately 30% amino acid identity with PiRV-3 hypothetical proteins and RdRp, respectively [17] while contig 3 (2957 nt) shared 31% amino acid identity with PiRV-4 [16].

**Figure 1:**
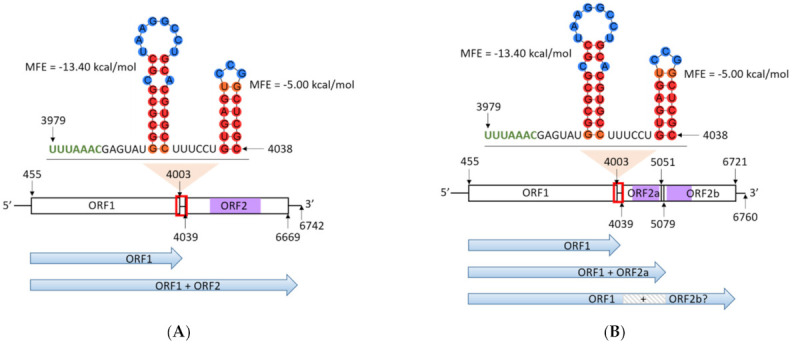
(**A**) Genome structure of Phytophthora pluvialis RNA virus 1 (PplRV1) genotype a from NZFS 3052. (**B**) Genome structure of PplRV1 genotype b from NZFS 4018. Horizontal bars represent open reading frames (ORFs). Purple bars represent RNA-dependent RNA polymerase (RdRp) regions where the conserved motifs are located. Lines represent the 5′ and 3′ ends and the gap sequence between ORF1 and ORF2. Nucleotide position in genomes are indicated by numbers. Red boxes indicate the position of the −1 reading frame signal sequence UUUAAAC (labeled in green), spacer region and downstream sequence with stem-loop structures. In the stem-loop structures, nucleotides in red/orange circular shades indicate high base-pairing probability. Nucleotides in blue circular shades indicate low base-pairing probability. Blue arrows represent possible protein translated from predicted ORFs. Grey box with diagonal lines indicates the protein that should be encoded by ORF2a. MFE: minimum free energy.

The presence of a single viral dsRNA genome, designated as Phytophthora pluvialis RNA virus 1 (PplRV1), was confirmed by RT-PCR. The complete PplRV1 reference genome sequence was obtained through Sanger sequencing using primer walking and 5′ RACE (Figure S1) from RNA extracted from isolate *P. pluvialis* NZFS 3052. The PplRV1_3052 genome is 6742 nt with a GC content of 55.4%, and two predicted ORFs. ORF1 is 3549 nt in length, starting at nt position 455 and terminating at 4003. A BLASTx analysis of the predicted ORF1 amino acid sequence showed similarity to hypothetical proteins found in other RNA viruses, including PiRV-3. The ORF2 is 2631 nt in length, starting at position 4039 and terminating at position 6669. The ORF2 is predicted to encode an RdRp, and contains six predicted motif regions. A 47 nt slippery sequence was predicted to span ORF1 and ORF2 from nt 3955 to nt 4001 (Figure 1A). A signal sequence for −1 translational frameshift, UUUAAAC, was identified immediately before the stop codon of ORF1. Moreover, a strong stem-loop structure forms in the slippery sequence and a six nt spacer region exists between the signal sequence and the stem-loop structure (Figure 1A). Together, these genomic signatures suggest that the two ORFs may be expressed as a fused protein.

To investigate the genetic diversity of PplRV1 in New Zealand *P. pluvialis* population, we isolated PplRV1 from a range of *P. pluvialis* isolates spanning both New Zealand clusters. Two distinct genotypes of PplRV1 (PplRV1a and b) were identified from the two host clusters (*P. pluvialis* NZ1 and NZ2) with complete correspondence, respectively. PplRV1 (genotype a) from isolate NZFS 3052 (*P. pluvialis* cluster NZ1) was chosen as the reference genome for the first genotype of the virus, and we propose to designate the virus genotype associated with NZFS 4018 (*P. pluvialis* cluster NZ2) as the reference genome for PplRV1b.

PplRV1_4018 has a genome size of 6760 nt with a GC content of 55.2%. Three ORFs including two sub-ORFs of ORF2 were identified in PplRV1_4018. ORF1 has a length of 3549 nt, starting at position 455 and terminating at 4003. ORF2a has a length of 1041 nt, which begins at position 4039 and ends at 5079. ORF2b is 1671 nt in length, from position 5051 to position 6721. Both ORF2a and ORF2b show similarity to parts of RdRp albeit encoded in two ORFs (Figure 1B). ORF2a and ORF2b contain different RdRp motifs, which are within six other PplRV1b isolates (refer to Section 3.3 for further details). Four PplRV1b isolates were sequenced that had the RdRp in a single ORF (ORF2). The separation of the RdRp into two ORFs is due to a change from a ‘GGG’ stretch to a ‘GGGG’ at position 5064 in PplRV1_4018. This insertion introduces a stop codon and terminates the predicted ORF.

PplRV1a and PplRV1b share 92.3% nt identity. Although PplRV1b has a different genome size and GC content from those of PplRV1a, it contains a perfectly conserved slippery sequence of the same size and in the same position and therefore identical stem-loops (Figure 1A,B).

### 3.2. PplRV1 Conserved Region and Related Viruses

**Figure 2:**
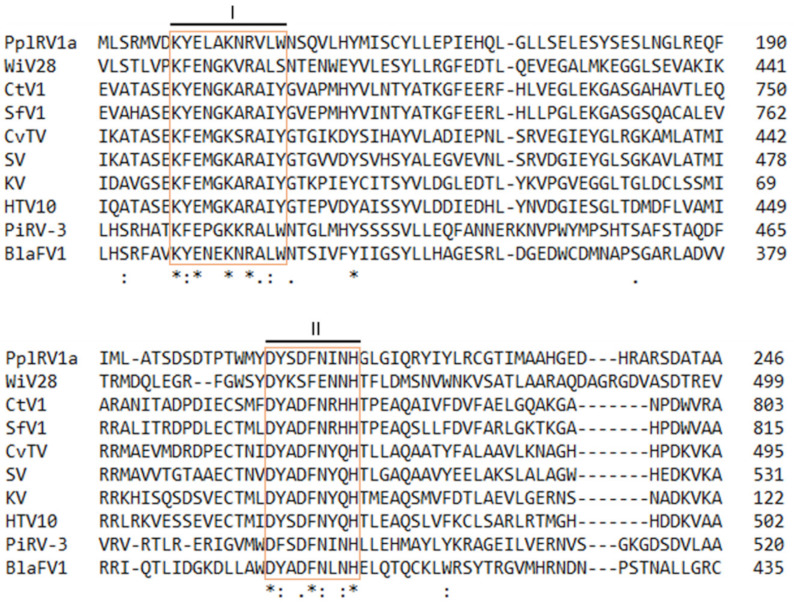

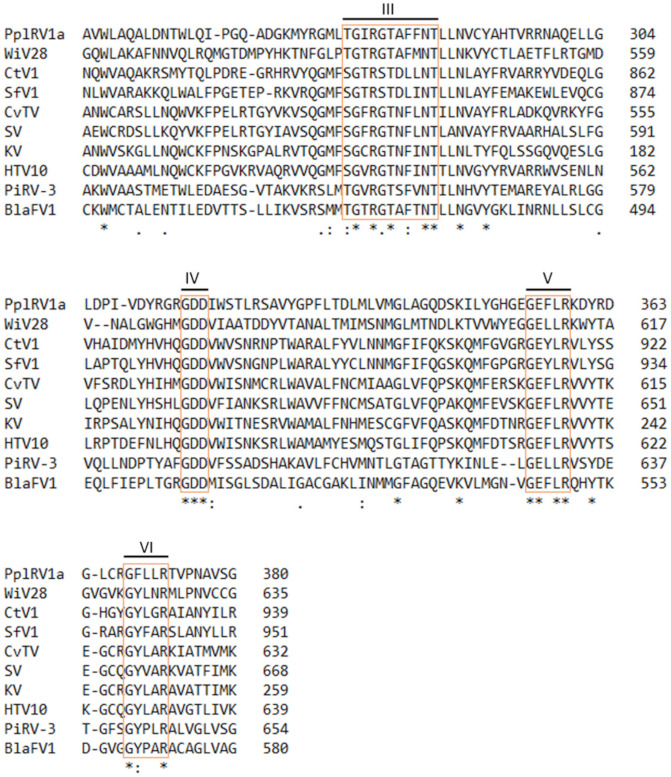
Alignment of amino acid sequence of Phytophthora pluvialis RNA virus 1 genotype a (PplRV1a) putative RNA-dependent RNA polymerase (RdRp) with the RdRp regions of viruses with predicted similarities. Orange boxes indicate the conserved motifs in the viral RdRps. Asterisk indicates positions which have a single, fully conserved residue. Colon indicates conservation between groups of strongly similar properties. Period indicates conservation between groups of weakly similar properties. Numbers indicate the length of amino acid sequence. WiV28: Wuhan insect virus 28 (accession: YP_009342430); CtV1: Circulifer tenellus virus 1 (accession: YP_003800003); SfV1: Spissistilus festinus virus 1 (accession: YP_003800001); CvTV: Culex vishnui subgroup totivirus (accession: BBQ05098); SV: Stinn virus (accession: QRW41701); KV: Koroku virus (accession: QRW41695); HTV10: Hubei toti-like virus 10 (accession: YP_009336493); PiRV-3: Phytophthora infestans RNA virus 3 (accession: YP_009551328); BlaFV1: Bremia lactucae associated fusagravirus1 (accession: QIP68009).

BLASTx searches using the protein sequence of the putative RdRp encoded by PplRV1a ORF2 found similarities with the RdRp regions of Wuhan insect virus 28 (WiV28), Circulifer tenellus virus 1 (CtV1), Spissistilus festinus virus 1 (SfV1), Culex vishnui subgroup totivirus (CvTV1), Stinn virus (SV), Koroku virus (KV), Hubei toti-like *virus 10* (HTV10), Phytophthora infestans RNA virus 3 (PiRV-3), and Bremia lactucae associated fusagravirus 1 (BlaFV1). Within these related viruses, CvTV belongs to *Totiviridae*, and CtV1, SfV1 and BlaFV1 are dsRNA viruses, while WiV28, and SV, KV HTV10 and PiRV3 are unclassified RNA viruses. PplRV1a RdRp has very low residue/base similarity percentage with these other viruses. The highest amino acid similarity is 32.64% with dsRNA virus, BLaFV1, and the lowest amino acid similarity is 24.94% with unclassified RNA virus, SV (Table 1). Alignment of PplRV1a putative RdRp with WiV28, CtV1, SfV1, CvTV, SV, KV, HTV10, PiRV-3 and BlaFV1 RdRps revealed some conserved residues present among these proteins (Figure 2). Fully conserved residues at amino acid positions are present in the motifs of PplRV1 putative RdRp (Figure 2). As typical in viral RdRps, there are several conserved motifs [36]. In PplRV1a, six conserved motifs exist in the putative RdRp, including motif I (KYELAKNRVLW), motif II (DYSDFNINH), motif III (TGIRGTAFFNT), motif IV (GDD), motif V (GEFLR) and motif VI (GFLLR) (Figure 2), while in totivirus CvTV, motif I is KFEMGKSRAIYG; motif II is DYADFNYQH; motif III is SGFRGTNFLNT; motifs IV and V are GDD and GEFLR, respectively, and motif VI is GYLAR (Figure 2). Except for motifs IV and V, other motifs in PplRV1a and CvTV are different. The lack of conservation of motifs I, II, III and VI of PplRV1 RdRp indicates the virus replication mechanism of PplRV1 may differ from that of the totivirus, CvTV. Overall, the conserved region and phylogenetic analysis indicate that PplRV1a differs from its related viruses. Therefore, PplRV1 is likely to be a member of a so far unclassified virus family.

### 3.3. PplRV1 Variants and P. pluvialis Cluster NZ1 and NZ2

PplRV1 from 23 *P. pluvialis* isolates were fully/near completely sequenced. Except PplRV1_3052 and PplRV1_4018, PplRV1 from NZFS 4015, NZFS 4016 and NZFS 4019 were also fully sequenced. The genome size of PplRV1_4015, PplRV1_4016 and PplRV1_4019 is 6758, 6758 and 6756 nt, respectively. The near-complete genomes of other PplRV1 genotypes are about 6500 nt (Table 2).

The data indicate two distinct PplRV1 variants associated with the two *P. pluvialis* New Zealand clusters. Comparison of the PplRV1 sequences purified from NZ1 *P. pluvialis* to the sequences isolated from NZ2 *P. pluvialis* showed the highest identity correlating with the *P. pluvialis* host cluster. NZ1-derived PplRV1 sequences are most like the NZ1 PplRV1 reference genome PplRV1a (97.8–99.1%) rather than the NZ2 PplRV1-derived reference genome PplRV1b (92.0–93.2%). Conversely, NZ2-derived PplRV1 sequences are most similarity to those of PplRV1b (99.2–99.5%) rather than PplRV1a (91.2–92.5%) (Table 2). The data indicate two distinct PplRV1 genotypes (a or b), each associated with only one of the two *P. pluvialis* New Zealand clusters (NZ1 or NZ2, respectively). The intra-variation and inter-variation between PplRV1 genotypes validate the link between the host clusters and virus genotypes.

**Figure 3:**
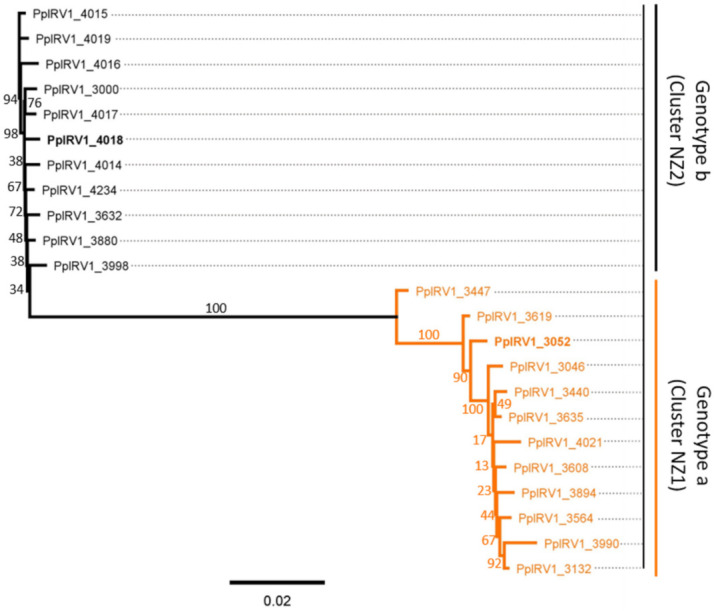
Maximum-likelihood phylogenetic tree based on the core genome alignment of Phytophthora pluvialis RNA virus 1 (PplRV1) sequences isolated from cluster NZ1 and NZ2 *Phytophthora pluvialis* isolates. The length of the core genome is 6512 nt, which was obtained by eight sequencing primer pairs (see Table S2 for details). Black color-coding viruses were isolated from cluster NZ2 *P. pluvialis* (PplRV1 genotype b). PplRV1_4018 is the reference for NZ2 *P. pluvialis* viruses. Orange color-coding viruses were isolated from cluster NZ1 *P. pluvialis* (PplRV1 genotype a). PplRV1_3052 is the reference for NZ1 *P. pluvialis* viruses. Numbers above the branches show bootstrap support in percentiles in 1000 replicates. Scale bar indicates substitutions per site.

A phylogenetic analysis also supports that NZ1-associated PplRV1a sequences and NZ2-associated PplRV1b sequences are clustered in two different groups, which has a 100% support for the grouping (Figure 3). In the PplRV1a group, several sub-groups are evident; the virus from NZFS 3477 is distinctive from other PplRV1a genotype viruses, as other PplRV1a genotype viruses are grouped in the same clade with 100% support. In the second class of the PplRV1a group, PplRV1_3619 is separated from other viruses, with a support of 90%. Furthermore, in the third class of the PplRV1a group, PplRV1_3052 has a 100% support for its grouping from other viruses. The rest of PplRV1a genotype viruses, PplRV1_3046, PplRV1_3440, PplRV1_3635, PplRV1_4021, PplRV1_3608, PplRV1_3894, PplRV1_3564, PplRV1_3990 and PplRV1_3132 cluster together, with a support of 17% (Figure 3). In the PplRV1b group, the sequence difference is smaller than in the PplRV1a genotype viruses, as the first sub-group with PplRV1_3998 alone has only 34% support for the clustering (Figure 3). The seven PplRV1b genomes with a split RdRp (PplRV1_4014, PplRV1_4015, PplRV1_4016, PplRV1_4017, PplRV1_4018, PplRV1_4019, and PplRV1_4234) do not aggregate together tightly in the phylogenetic tree and are distributed amongst the other four genotype b isolates that are predicted to have a single RdRp ORF. Despite the presence of an additional nucleotide resulting in putative split RdRp in seven of the eleven PplRV1b genomes, the distribution of the PplRV1a and PplRV1b variants exactly mirrors the geographical distribution of the host *P. pluvialis* in New Zealand.

### 3.4. Diagnostics of PplRV1 in Phytophthora

To facilitate further studies of PplRV1, a qPCR diagnostic for the rapid detection and quantification of the virus was developed. Based on the genome comparisons, the ORF1 region of PplRV1_4018 was chosen as a target as it is the common part of the virus genome, and generic for both PplRV1a and PplRV1b genotype viruses. Furthermore, a multiplex assay that targets the *Phytophthora trnM-trnP-trnM* region was developed [37]. This host gene target is specific for the genus *Phytophthora* and allows the simultaneous detection and quantification of the host.

Using a plasmid bearing the target sequences, the limit for virus detection was determined as below 10 copies, and the limit for host detection was established as fewer than 100 copies. The specificity and sensitivity of the assay were established using a panel of 35 *Phytophthora*/*Nothophytophthora* isolates (Table S4). Of these, 25 were *P. pluvialis* bearing PplRV1, and ten were other *Phytophthora*/*Nothophytophthora* species not bearing the virus. According to this panel, the specificity and the sensitivity were both 100%, meaning that across the panel no false positives or false negatives were observed.

## 4. Discussion

### 4.1. Brief Summary of Findings

A novel RNA virus designated as PplRV1 was isolated from *P. pluvialis* in New Zealand. The PplRV1 reference genome encodes two putative proteins, a hypothetical protein and an RdRp. No homology to virus encoded coat proteins was identified within the PplRV1 genome. There are two distinct PplRV1 genotypes: the genotype associated exclusively with *P. pluvialis* cluster 1 designated as PplRV1a, and the genotype associated exclusively with *P. pluvialis* cluster 2, PplRV1b. PplRV1a RdRp is predicted to be encoded by a single protein, while the reference genome for the PplRV1b genotype (PplRV1_4018) has the putative RdRp split between ORF2a and ORF2b that is conserved in seven of eleven genotype b isolates. The qPCR multiplex diagnostic developed for the rapid detection and quantification of both the virus and host revealed that all *P. pluvialis* isolates tested (n = 25) were infected with PplRV1.

### 4.2. Further Investigation of Genomic Diversity

As reported previously, the genetic diversity of *P. pluvialis* population shows that *P. pluvialis* cluster 1 (carrying PplRV1a) was the initial introduction into New Zealand [10]. Although the genome of *P. pluvialis* cluster 2 is diverse from that of cluster 1, the phylogenetic analysis revealed that all *P. pluvialis* cluster 1 and cluster 2 isolates were grouped in a single terminal clade, which indicates there was only a single incursion of *P. pluvialis* into New Zealand [10]. Since RNA virus replication has high mutation rates, dynamic mutant swarms, known as “viral quasispecies” can form within the host population [38]. PplRV1 may have diverged within New Zealand. This PplRV1 genetic diversity may maintain viral fitness to adapt to genomic variations of its host or the environment, which could explain why some PplRV1b isolates have their putative RdRp split between ORF2a and ORF2b as depicted in Figure 1 for PplRV1_4018.

A −1 frameshifting potentially occurs in a “slippery” overlap sequence between ORF1 and ORF2 in PplRV1. Two stem-loop structures were also predicted downstream of this slippery sequence, which supports the possibility of ribosomal frameshifting in the progress of PplRV1 polyprotein translation. Stem-loops relevant to slippery sequences are also known as “stimulatory” RNA secondary structures, and are able to modulate the −1 frameshift efficiency [39]. Therefore, the stem-loop structures in slippery sequence may play an important role in the control of PplRV1 replication efficiency. For PiRV-3, an in-depth analysis of the translational scenario showed that a slippery sequence caused a −1 frameshift between ORF1 and ORF2, thereby enabling the expression of a fusion protein [17]. The structure of PplRV1_3052 (genotype a) points to an analogous mechanism that allows either the translation of an ORF1-ORF2 fusion protein, or additionally the possibility of translation of ORF1 by itself. In PplRV1_4018 (and several other genotype b isolates), the situation is less clear, owing to a single nucleotide insertion at position 5064 within ORF2. The described mechanism of a −1 frameshift would in this case lead to an ORF1-ORF2a fusion protein. ORF2b, on the other hand, is encoded in the same reading frame as ORF1, and contains several of the critical residues for RdRp function. It remains unclear if a regulation of the −1 frameshift would allow the translation of an ORF1-ORF2b fusion protein, which then complements an ORF1-ORF2a fusion protein into a functional RdRp, or if a different, so far unidentified mechanism allows the translation of this ORF. Additional research is necessary to elucidate this enigma, and to understand its evolutionary history and impact.

A BLAST search using the PplRV1 ORF2 sequence revealed that it has similarity to PiRV-3, totiviruses and the RdRps of unclassified RNA viruses. However, phylogenetic analysis of PplRV1 RdRps and these related viral RdRps found PplRV1 does not cluster with any of those viruses (Table 1). Moreover, protein alignment between the RdRps of PplRV1 and its relevant viruses found PplRV1 shares very low similarity with them (Table 1). In fact, all other viruses are more closely related to each other than any of them is to PplRV1. Therefore, PplRV1 may represent the first member of a new RNA virus family.

### 4.3. qPCR Multiplex Diagnostic

In this study, a diagnostic method using qPCR technique has been developed, which can accurately and quantitatively detect PplRV1 and *Phytophthora* using a single sample. Compared with the traditional diagnostic method of using standard PCR, our diagnostics can be used to determine the titers of PplRV1 by using an appropriate standard. For that purpose, the target regions of the assay were placed on a plasmid, and appropriate dilutions were created to represent specific copy numbers.

We detected PplRV1 exclusively in *P. pluvialis* and not in other *Phytophthora*/*Nothophytophthora* species pure cultures so far, despite ten isolates being from other species. It would be beneficial to investigate a broader variety of *Phytophthora* species and other oomycete species as well as environmental samples, i.e., infected pine needles, and water and soil samples from pine woods. A comprehensive survey of PplRV1 in isolates from North America could shed light on the biology of PplRV1, and potentially on the history of its introduction to New Zealand. With the help of PplRV1 diagnostics described in this work, we can obtain a better understanding of the prevalence and host range of PplRV1.

### 4.4. PplRV1 Distribution

Investigation of PplRV1 (a and b) distribution correlated entirely with the *P. pluvialis* cluster (1 and 2, respectively), indicating co-evolution of PplRV1 with its host *P. pluvialis* cluster since its introduction to New Zealand [6]. The result also implies low mobility of PplRV1 between collocated *P. pluvialis* isolates. The lack of PplRV1 movement between host clusters could be caused by exclusion (i.e., all *P. pluvialis* in New Zealand carry the virus and an already infected isolate cannot be superinfected by a different virus variant), a lack of a vector for PplRV1 in New Zealand, or the inability for PplRV1 to move by anastomosis between the two *P. pluvialis* clusters present in New Zealand. It is unlikely that PplRV1 forms a virion and the unencapsulated RNA may be unstable in the environment. Whether the initial introduction of *P. pluvialis* carried PplRV1, or if this association occurred shortly after entry into New Zealand, will require further research to confirm. Importantly, research is needed to survey the distribution of PplRV1 in North America, and to determine its genomic features. In addition, determining whether PplRV1 is present in other *Phytophthora* species within New Zealand, the USA and other countries, especially those growing *Pseudotsuga menziesii* and *Pinus radiata,* would provide a clearer picture of its worldwide distribution and introduction history into New Zealand.

### 4.5. Future Research

The unique genetic structure of some PplRV1b isolates leads to additional questions, for example, how are ORF2a and ORF2b expressed to form a functional RdRp enzyme? A thorough biochemical investigation of the RdRp enzymes from isolates with the different genome structures could illuminate the function and potential evolutionary relevance of the different genome organizations described in this work.

An analysis of the presence of PplRV1 in *P. pluvialis* strains isolated in North America, and the comparison of their genomes with the ones described here, will provide further insight into the function of the virus.

The significance of PplRV1a and b for its host remains unclear. To date, no *P. pluvialis* isolate without one of the PplRV1 virus variants has been observed. The generation of such a PplRV1-free isolate will be crucial for future work to understand the ecological and evolutionary roles of the virus.

Some mycoviruses affect the host virulence and may play an important role in the development of the controlling methods for plant diseases. For example, CHV1 decreases the pathogenicity of its host *C. parasitica* which was used as a biocontrol agent of chestnut blight in Europe [21]; Conversely, PiRV-2 stimulates the sporangia production in *P. infestans* and could impact the ecological fitness of *P. infestans* [23]. Since *P. pluvialis* results in severe red needle cast disease in New Zealand, future research on the virus transmission and whether PplRV1 affects host virulence (i.e., hyper- or hypo-virulence) will be important for the development of a potential biocontrol for red needle cast disease.

## Figures and Tables

**Table 1:** Percentage identity of residues/bases among putative RNA-dependent RNA polymerase (RdRp) of Phytophthora pluvialis RNA virus 1 genotype a (PplRV1a) and RdRps of Culex vishnui subgroup totivirus (CvTV), Bremia lactucae associated fusagravirus1 (BlaFV1), Koroku virus (KV), Stinn virus (SV), Spissistilus festinus virus 1 (SfV1), Circulifer tenellus virus 1 (CtV1), Hubei toti-like virus 10 (HTV10), Wuhan insect virus 28 (WiV28) and Phytophthora infestans RNA virus 3 (PiRV-3).

	PplRV1a	CvTV	BlaFV1	KV	SV	SfV1	CtV1	HTV10	WiV28	PiRV-3
CvTV	25.97									
BlaFV1	32.64	24.23								
KV	27.65	57.14	26.79							
SV	24.94	69.42	24.74	53.28						
SfV1	25.42	34.15	23.56	44.23	32.93					
CtV1	26.09	35.04	20.62	43.08	34.31	62.29				
HTV10	25.71	55.32	24.23	60.62	53.19	35.85	34.55			
WiV28	27.62	25.77	24.37	30.22	22.96	24.05	23.52	22.70		
PiRV-3	29.72	24.68	31.78	24.72	24.94	19.19	19.14	26.74	24.87	

**Table 2:** A list of information of Phytophthora pluvialis RNA virus 1 genotype a (PplRV1a) and Phytophthora pluvialis RNA virus 1 genotype b (PplRV1b) sequences obtained from *P. pluvialis* cluster NZ1 and NZ2 isolates, respectively. Viral sequences were obtained by primer walking and dsRNA sequencing. PplRV1_3052 and PplRV1_4018 are the reference for PplRV1a and PplRV1b genotype viruses, respectively. The full length of reference genomes was determined by 5′ rapid amplification of cDNA ends (5′ RACE). Viruses were named corresponding to their *P. pluvialis* host names. For example, PplRV1_3052 was derived from the *P. pluvialis* isolate NZFS 3052.

Virus Name	GenBank Accession	Genotype	Length (nt)	GC%	Number of Predicted RdRp ORFs	Identical Sites Compared with PplRV1_4018	Identical Sites Compared with PplRV1_3052
PplRV1_3052	OL799269	PplRV1a	6742 *	55.4	1	92.3%	-
PplRV1_3046	OL799282	PplRV1a	6508	55.7	1	92.0%	99.0%
PplRV1_3132	OL799274	PplRV1a	6529	55.6	1	92.2%	98.9%
PplRV1_3440	OL799283	PplRV1a	6496	55.8	1	92.0%	98.9%
PplRV1_3447	OL799284	PplRV1a	6604	55.4	1	93.2%	97.8%
PplRV1_3564	OL799285	PplRV1a	6578	55.7	1	92.1%	98.7%
PplRV1_3608	OL799275	PplRV1a	6541	55.7	1	92.1%	98.9%
PplRV1_3619	OL799286	PplRV1a	6583	55.6	1	92.3%	99.1%
PplRV1_3635	OL799288	PplRV1a	6544	55.6	1	92.1%	99.1%
PplRV1_3894	OL799276	PplRV1a	6512	55.6	1	92.2%	98.9%
PplRV1_3990	OL799290	PplRV1a	6566	55.7	1	92.0%	98.2%
PplRV1_4021	OL799291	PplRV1a	6564	55.7	1	92.2%	98.8%
PplRV1_4018	OL799270	PplRV1b	6760 *	55.2	2	-	92.3%
PplRV1_3000	OL799281	PplRV1b	6597	55.6	1	99.4%	92.3%
PplRV1_3632	OL799287	PplRV1b	6557	55.6	1	99.4%	92.4%
PplRV1_3880	OL799289	PplRV1b	6563	55.6	1	99.5%	92.5%
PplRV1_3998	OL799277	PplRV1b	6545	55.6	1	99.2%	92.4%
PplRV1_4014	OL799278	PplRV1b	6677	55.5	2	99.4%	92.4%
PplRV1_4015	OL799271	PplRV1b	6758 *	55.3	2	99.5%	92.3%
PplRV1_4016	OL799272	PplRV1b	6758 *	55.2	2	99.3%	92.2%
PplRV1_4017	OL799279	PplRV1b	6658	55.6	2	99.5%	91.2%
PplRV1_4019	OL799273	PplRV1b	6756 *	55.3	2	99.3%	92.2%
PplRV1_4234	OL799280	PplRV1b	6554	55.6	2	99.5%	92.5%

* Full length as determined through 5′ RACE.

## Data Availability

Data supporting reported results are available from GenBank under accession numbers listed in Table 2.

## Supplementary Materials

The following supporting information can be downloaded at: https://www.mdpi.com/article/10.3390/v14020247/s1, Figure S1: Agarose-gel electrophoresis of PplRV1_4018 dsRNA RT-PCR products; Table S1: *Phytophthora pluvialis* isolates used for virus genome characterisation in this study; Table S2: A list of primers used for Phytophthora pluvialis RNA virus 1 (PplRV1) dsRNA sequencing and PplRV1 detection and diagnostics; Table S3: The optimized reaction of RT-qPCR for Phytophthora pluvialis RNA virus 1 detection; Table S4: A panel of *Phytophthora*/*Nothophytophthora* isolates used for the virus screen, including 25 *Phytophthora pluvialis* isolates, nine other *Phytophthora* species/isolates and one *Nothophytophthora* species.
