# Supplementary material for: Characterization of a Novel Double-Stranded RNA Virus from Phytophthora pluvialis in New Zealand
Source: Viruses. 2022 Jan 26;14(2):247. doi: 10.3390/v14020247 (PMC8878296; doi:10.3390/v14020247)
Supplement: Supplementary file 1 [file viruses-14-00247-s001.zip › viruses-1532661-supplementary.pdf]

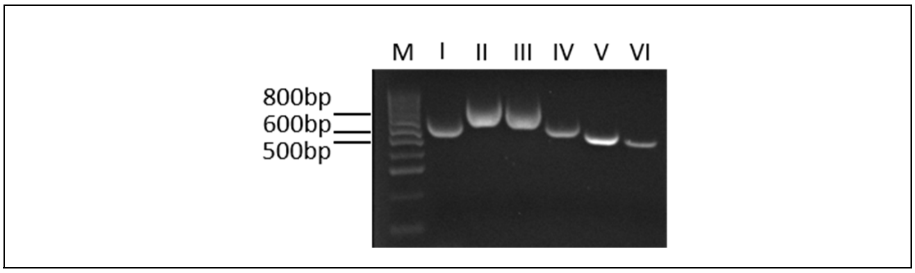

Figure S1. Agarose-gel electrophoresis of PplRV1_4018 dsRNA RT-PCR products. M: 1kb ladder; I-VI: RT-PCR amplicons obtained by primer walking.

Table S1. *Phytophthora pluvialis* isolates used for virus genome characterisation in this study. All *P. pluvialis* isolates were sourced from Scion (Rotorua, New Zealand).

Isolate	Cluster[1]	Geographical location	Host
NZFS 3000	NZ2	Gisborne	*Pinus radiata*
NZFS 3046	NZ1	Northland	*Pinus radiata*
NZFS 3052	NZ1	Northland	*Pinus radiata*
NZFS 3132	NZ1	Coromandel	*Pinus radiata*
NZFS 3440	NZ1	Taupo	*Pinus radiata*
NZFS 3447	NZ1	Nelson	*Pinus radiata*
NZFS 3564	NZ1	Hawke's Bay	*Pinus radiata*
NZFS 3608	NZ1	Northland	*Pinus radiata*
NZFS 3619	NZ1	Nelson	*Pinus radiata*
NZFS 3632	NZ2	Bay of Plenty/Wanganui	*Pinus radiata*
NZFS 3635	NZ1	Nelson	*Pinus radiata*
NZFS 3880	NZ2	Wairarapa	*Pseudotsuga menziesii*
NZFS 3894	NZ1	Northland	*Pinus radiata*
NZFS 3990	NZ1	Auckland	*Pinus radiata*
NZFS 3998	NZ2	Coromandel	*Pinus radiata*
NZFS 4014	NZ2	Gisborne	*Pinus radiata*
NZFS 4015	NZ2*	Gisborne	*Pinus radiata*
NZFS 4016	NZ2	Gisborne	*Pinus radiata*
NZFS 4017	NZ2*	Gisborne	*Pinus radiata*
NZFS 4018	NZ2	Gisborne	*Pinus radiata*
NZFS 4019	NZ2	Gisborne	*Pinus radiata*
NZFS 4021	NZ1	Northland	*Pinus radiata*
NZFS 4293	NZ1	Taranaki	*Pinus radiata*

[1] The cluster nomenclature was according to Tibima et al. (2021).

*Isolate classification was based on the virus phylogenetic analysis referred to in **Figure 3**.

Table S2. A list of primers used for Phytophthora pluvialis RNA virus 1 (PplRV1) dsRNA sequencing and PplRV1 detection and diagnostics.

**Primer name**	**Sequence (5' – 3')**	**Position (nt)**	**Purpose**
SEQ31[1]	CCATCGTCTTGGCCTATG	31-48	Sequencing primer, detection primer
SEQ997[1]	GATTCGTCTCGATGTCCC	1,014-997	Sequencing primer
SEQ718[1]	CGCGACAGAGTTTTTACG	718-735	Sequencing primer
SEQ1566[1]	GGTCTTCGATACGACACG	1,583-1,566	Sequencing primer
SEQ903[1]	GTGGTTGGACACACCCCG	903-920	Sequencing primer
SEQ1980[1]	TACCAGAAAGCCTCAGC	1,996-1,980	Sequencing primer
SEQ1561[1]	CTAACCGTGTCGTATCG	1,561-1,577	Sequencing primer
SEQ2786[1]	CCATGCCGAACTGGATAC	2,803-2,786	Sequencing primer
SEQ1702[2]	CGCTAATGGGCTAGACAC	1,702-1,719	Sequencing primer
SEQ3147[2]	GGGCGATCAGAGTGAATC	3,147-3,130	Sequencing primer
SEQ2679[1]	CCTCATCTCATCGCTCCACC	2,679-2,698	Sequencing primer
SEQ3504[1]	GCCATGGGTAGAAACTGG	3,520-3,504	Sequencing primer
SEQ3018[1]	AACTTCGTCGAGCCACG	3,018-3,034	Sequencing primer
SEQ4120[1]	GTGTACCCATGAACGCCCTC	4,139-4,120	Sequencing primer
SEQ4029[1]	GTGCCTTTCCTGTGAGTC	4,029-4,046	Sequencing primer
SEQ5136[1]	AGCCAGCATGATGAACTG	5,153-5,136	Sequencing primer
SEQ4664[2]	TGCATACTGACCCAACACTG	4,683-4,664	Sequencing primer
SEQ5210[1]	CCACGGTTTAGGTATCCAGC	5,210-5,229	Sequencing primer
SEQ5877[1]	TACAGAGTGACATGTCCTGC	5,895-5,877	Sequencing primer
SEQ4269[2]	CCAACATACGCGCGGGCG	4,269-4,286	Sequencing primer
SEQ4961[2]	ACCATCCGACTCAGCATTTC	4,980-4,961	Sequencing primer
SEQ4606[2]	TGATTACGCAACCCAACC	4,606-4,623	Sequencing primer
SEQ5928[2]	ATTCAAACCGGGTCCACCTC	5,947-5,928	Sequencing primer
SEQ5495[2]	ATCGTCGATTACCGTGGTCG	5,495-5,514	Sequencing primer

SEQ6663[2]	CAATGACCCGTACAGTCG	6,680-6,663	Sequencing primer, detection primer
RACE1	GATGGCAGCAGTAGTGTA	457-440	5’RACE primer
RACE2	CACTACTCGGACTGTTTC	542-525	5’RACE primer
RACE3	TCTCCAGGAGCACACCGGGC	219-200	5’RACE primer
RACE4	CGTGTGCTATGCTCATAC	5,454-5,437	5’RACE primer
RACE5	TGTTAGTGATGGGTCTGG	5,594-5,577	5’RACE primer
RACE6	GCAAGTGTGCAGACGCGGCG	5,873-5,854	5’RACE primer
PplRV1_qPCR_F1	GAACCCGAAACAGTCCGAGT	434-453	Diagnostic primer
PplRV1_qPCR_R1	AGAACCCACCGAAGTTAGCG	596-577	Diagnostic primer
PplRV1_probe1[5]	TGCTGCCATCCTCGTTTTCTTCGCG	533-557	Diagnostic probe
PplRV1_qPCR_F2	GCTCGGTTGTCGGTTTTTCC	4,186-4,205	Diagnostic primer
PplRV1_qPCR_R2	AGACCACATTTCGCAGCGTA	4,328-4,309	Diagnostic primer
PplRV1_qPCR_F3	GATACCCGGACAGGCTGATG	5,366-5,385	Diagnostic primer
PplRV1_qPCR_R3	CGACCACGGTAATCGACGAT	5,527-5,508	Diagnostic primer
[3,4]PhyG-F2	CGTGGGAATCATAATCCT	7,524-7,541	Diagnostic primer
[3,4]PhyG-Rb	CAGATTATGAGCCTGATAAG	7,745-7,726	Diagnostic primer
[3,4]TrnM_PhyG_probe2[6]	ATRTTGTAGGTTCAARTCCTAYCATCAT	7,543-7,570	Diagnostic probe
[3,4]PhyP_ATP9_F1	AGTAAGTATTTACGTATCCA	28,465-28,484	Diagnostic primer
[3,4]PhyP_ATP9_R1	GAAATCCTTCTTTACAACAAG	28,654-28,634	Diagnostic primer

[1]Primer was used for NZ1 PplRV1 dsRNA sequencing. Primer position is given relative to the reference PplRV1_4018.

[2]Primer was used for NZ2 PplRV1 dsRNA sequencing. Primer position is given relative to the reference PplRV1_3052.

[3]Primers and probe are originated from Bilobeau et al. (2014).

[4]Primer position is given relative to the reference NZFS3000 *P. pluvialis* mitogenome.

[5]The probe was labeled with the fluorescent dye FAM.

[6]The probe was labeled with the fluorescent dye Quasar 705.

Table S3. The optimized reaction of RT-qPCR for Phytophthora pluvialis RNA virus 1 detection. The RT-qPCR targets both virus and host sequences in a single reaction. Details of primers and probes are listed in **Table S2**.

	Component	Volume per 20 µL reaction
	iTaq Universal Probes rxn Mix (2×)	10 µL
	iScript Reverse Transcriptase	0.5 µL
Virus	10 µM PplRV1_qPCR_F1	0.8 µL (400 nM)
	10 µM PplRV1_qPCR_F1	0.8 µL (400 nM)
	10 µM PplRV1_probe1	0.5 µL (250 nM)
Host	10 µM PhyG-F2	1 µL (500 nM)
	10 µM PhyG-Rb	1 µL (500 nM)
	10 µM TrnM_PhyG_probe2	0.5 µL (250 nM)
	Nuclease-free H_2O	2.9 µL
	RNA template	2 µL

Table S4. A panel of *Phytophthora/Nothophytophthora* isolates used for the virus screen, including 25 *Phytophthora pluvialis* isolates, nine other *Phytophthora* species/isolates and one *Nothophytophthora* species. NZFS isolates were sourced from Scion (Rotorua, New Zealand). International Collection of Microorganisms from Plants (ICMP) isolates were sourced from Manaaki Whenua – Landcare Research (Auckland, New Zealand).

Isolate	Species	Geographical location	Host
ICMP 19002	*P. multivora*	Auckland	*Sophora* spp.
ICMP 21309	*P. amnicola × chlamydospora*	Auckland	Unknown
ICMP 21668	*P. cryptogea*	South Canterbury	*Hebe.* spp.
ICMP 21670	*P. cryptogea*	Auckland	*Agathis australis*
ICMP 21671	*P. multivora*	Bay of Plenty	*Araucaria heterophylla*
NZFS 119.03	*P. cactorum*	Bay of Plenty	*Meryta sinclaririi*
NZFS 3859	*P. cactorum*	Bay of Plenty	*Diospryros* sp.
NZFS 3003	*P. pluvialis*	Rangitikei	*Pinus radiata*
NZFS 3092	*P. pluvialis*	Waikato	*Pinus radiata*
NZFS 3565	*P. pluvialis*	Hawke's Bay	*Pinus radiata*
NZFS 3612	*P. pluvialis*	Hawke's Bay	*Pinus radiata*
NZFS 3618	*P. pluvialis*	Wellington	*Pinus radiata*
NZFS 3620	*P. pluvialis*	Rangitikei	*Pinus radiata*
NZFS 3689	*P. pluvialis*	Wanganui	*Pinus radiata*
NZFS 3690	*P. pluvialis*	Rangitikei	*Pinus radiata*
NZFS 3697	*P. pluvialis*	Gisborne	*Pseudotsuga menziesii*
NZFS 3701	*P. pluvialis*	Hawke's Bay	*Pseudotsuga menziesii*
NZFS 3859	*P. cactorum*	Bay of Plenty	*Diospryros* sp.
NZFS 3881	*P. pluvialis*	Wairarapa	*Pseudotsuga menziesii*

NZFS 3882	*P. pluvialis*	Wairarapa	*Pseudotsuga menziesii*
NZFS 3896	*P. pluvialis*	Wellington	*Pinus radiata*
NZFS 3897	*P. pluvialis*	Wellington	*Pinus radiata*
NZFS 3963	*P. pluvialis*	Nelson	*Pseudotsuga menziesii*
NZFS 3987	*P. pluvialis*	Bay of Plenty	*Pinus radiata*
NZFS 3993	*P. pluvialis*	Nelson	*Pseudotsuga menziesii*
NZFS 4001	*P. pluvialis*	Nelson	*Pseudotsuga menziesii*
NZFS 4002	*P. pluvialis*	Nelson	*Pseudotsuga menziesii*
NZFS 4020	*P. pluvialis*	Bay of Plenty	*Pinus radiata*
NZFS 4034	*P. pluvialis*	Nelson	*Pinus radiata*
NZFS 4045	*P. pluvialis*	Taupo	*Pinus radiata*
NZFS 4171	*P. pluvialis*	Taupo	*Pinus radiata*
NZFS 4273	*P. pluvialis*	Taupo	*Pinus radiata*
NZFS 4293	*P. pluvialis*	Taranaki	*Pinus radiata*
NZFS 4311	*P. amnicola × chlamydospora*	Bay of Plenty	Unknown
NZFS 4487	*Nothophytophthora* sp.	Unknown	Unknown
NZFS 4591	*P. amnicola × themophila*	Northland	*Pinus radiata*
